# Supplementary material for: Pharmacological Evaluation of Newly Synthesized Cannabidiol Derivates on H9c2 Cells
Source: Antioxidants (Basel). 2023 Sep 4;12(9):1714. doi: 10.3390/antiox12091714 (PMC10525859; doi:10.3390/antiox12091714)
Supplement: Supplementary file 1 [file antioxidants-12-01714-s001.zip › antioxidants-2563307-supplementary.pdf]

Supporting Information

Pharmacological Evaluation of Newly Synthesized Cannabidiol Derivates on H9c2 Cells

Kitti Szőke [1,2], Richárd Kajtár [1], Alexandra Gyöngyösi [1], Attila Czompa [1], Adina Fésüs [1,2], Eszter Boglárka Lőrincz [3,4], Ferenc Dániel Petróczi [3,4], Pál Herczegh [3], István Bak [1], Anikó Borbás [3], Ilona Bereczki [3,5,6,*] and István Lekli [1,*]

1 Department of Pharmacology, Faculty of Pharmacy, University of Debrecen, 4032 Debrecen, Hungary; szoke.kitti@pharm.unideb.hu (K.S.); kajtar.ricsi@gmail.com (R.K.); gyongyosi.alexandra@pharm.unideb.hu (A.G.); czompa.attila@gmail.com (A.C.); fesus.adina@pharm.unideb.hu (A.F.); bak.istvan@pharm.unideb.hu (I.B.)
2 Institute of Healthcare Industry, University of Debrecen, 4032 Debrecen, Hungary
3 Department of Pharmaceutical Chemistry, Faculty of Pharmacy, University of Debrecen, 4032 Debrecen, Hungary; lorincz.eszter@pharm.unideb.hu (E.B.L.); petroczi.f.daniel@gmail.com (F.D.P.); herczegh.pal@pharm.unideb.hu (P.H.); borbas.aniko@science.unideb.hu (A.B.)
4 Doctoral School of Pharmaceutical Sciences, University of Debrecen, 4032 Debrecen, Hungary
5 National Laboratory of Virology, Szentágothai Research Centre, 7624 Pécs, Hungary
6 ELKH-DE Pharmamodul Research Team, University of Debrecen, 4032 Debrecen, Hungary
* Correspondence: bereczki.ilona@pharm.unideb.hu (I.B.); lekli.istvan@pharm.unideb.hu (I.L.); Tel.: +36-52-255-586 (I.L.)

Citation: Szőke, K.; Kajtár, R.; Gyöngyösi, A.; Czompa, A.; Fésüs, A.; Lőrincz, E.B.; Petróczi, F.D.; Herczegh, P.; Bak, I.; Borbás, A.; et al. Pharmacological Evaluation of Newly Synthesized Cannabidiol Derivates on H9c2 Cells. *Antioxidants* **2023**, *12*, 1714. https://doi.org/10.3390/antiox12091714

Academic Editor: Sonia Trombino

Received: 1 August 2023
Revised: 25 August 2023
Accepted: 31 August 2023
Published: 4 September 2023

Copyright: © 2023 by the authors. Submitted for possible open access publication under the terms and conditions of the Creative Commons Attribution (CC BY) license (https://creativecommons.org/licenses/by/4.0/).

Supporting Information

The NMR spectra for the newly synthesized compounds are given below.

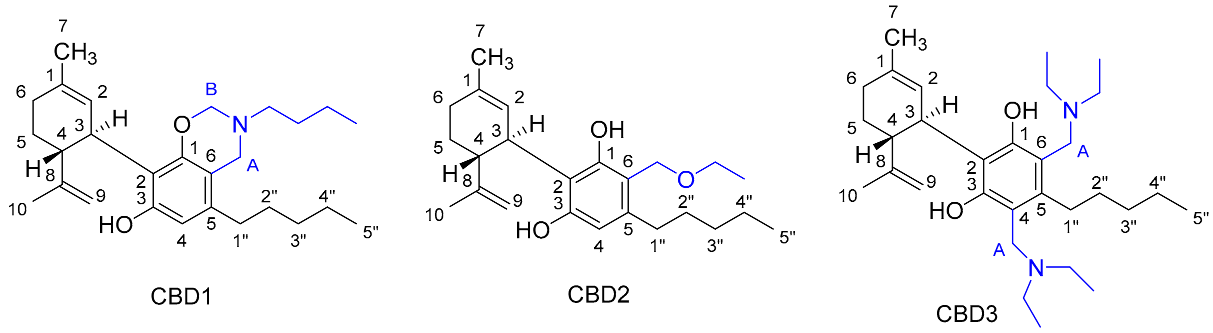

Figure S1. Numbering of the synthesized derivatives.

The NMR spectra for the newly synthesized compounds are given below.

NMR spectra of CBD1

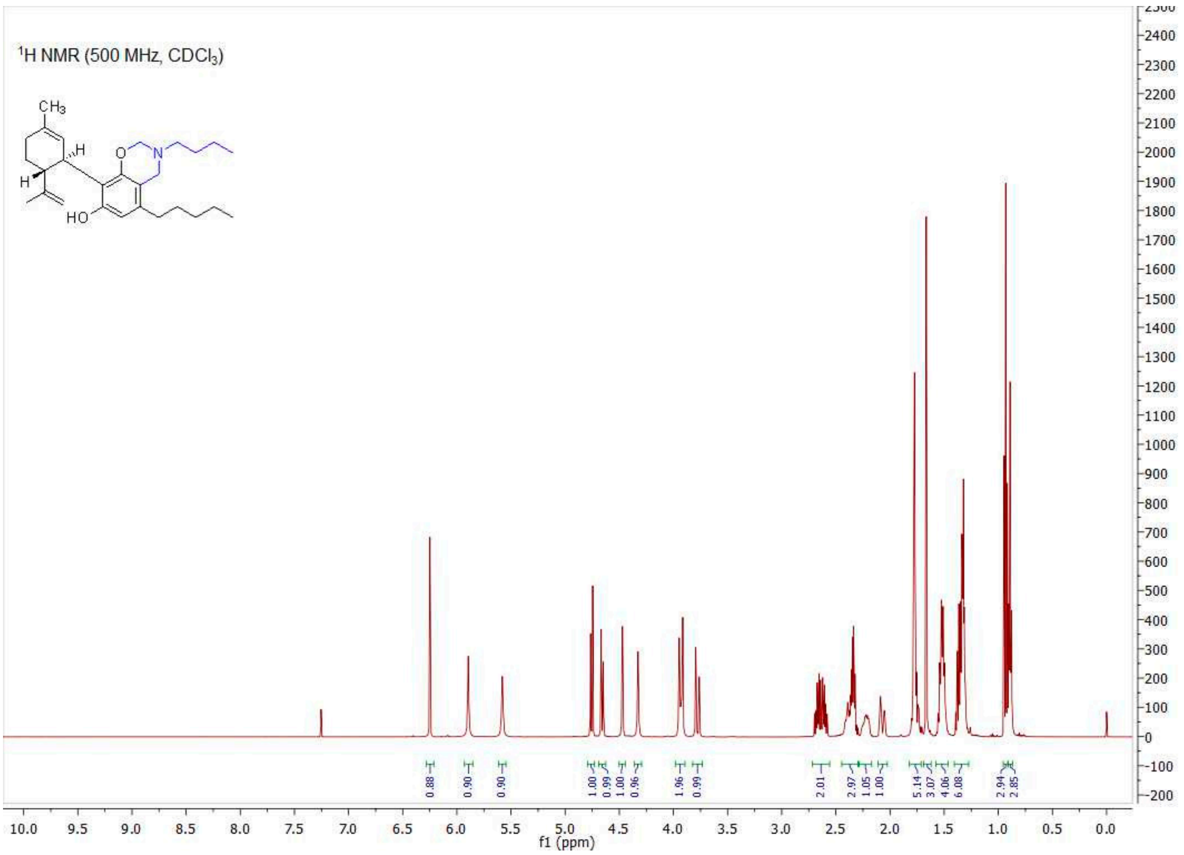

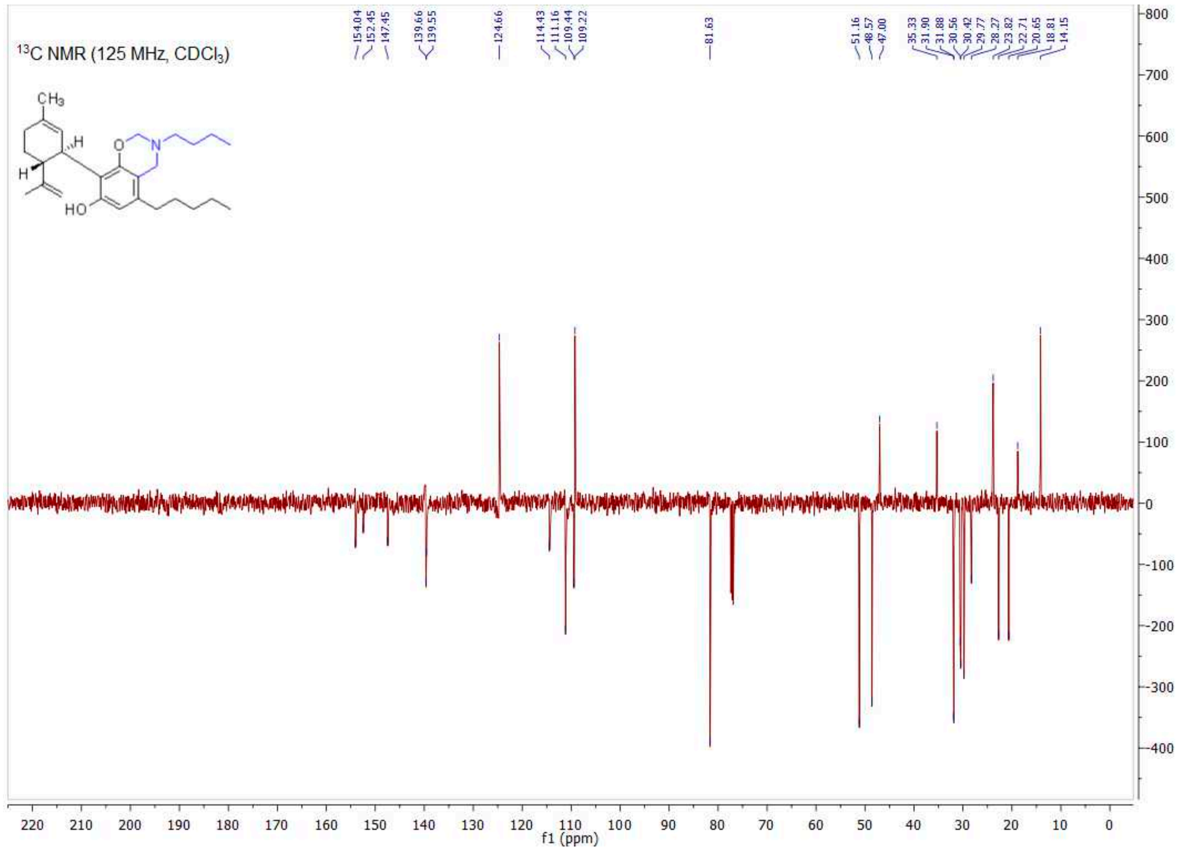

NMR spectra of CBD2

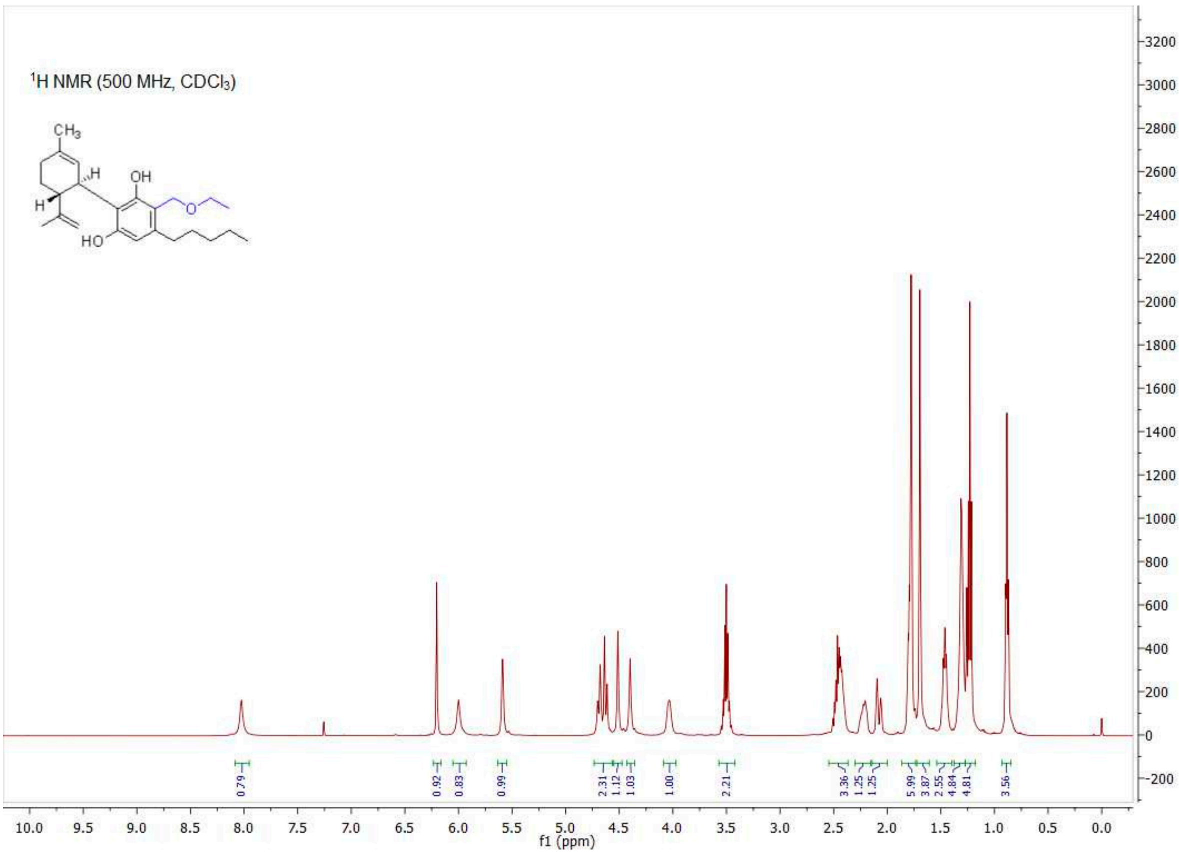

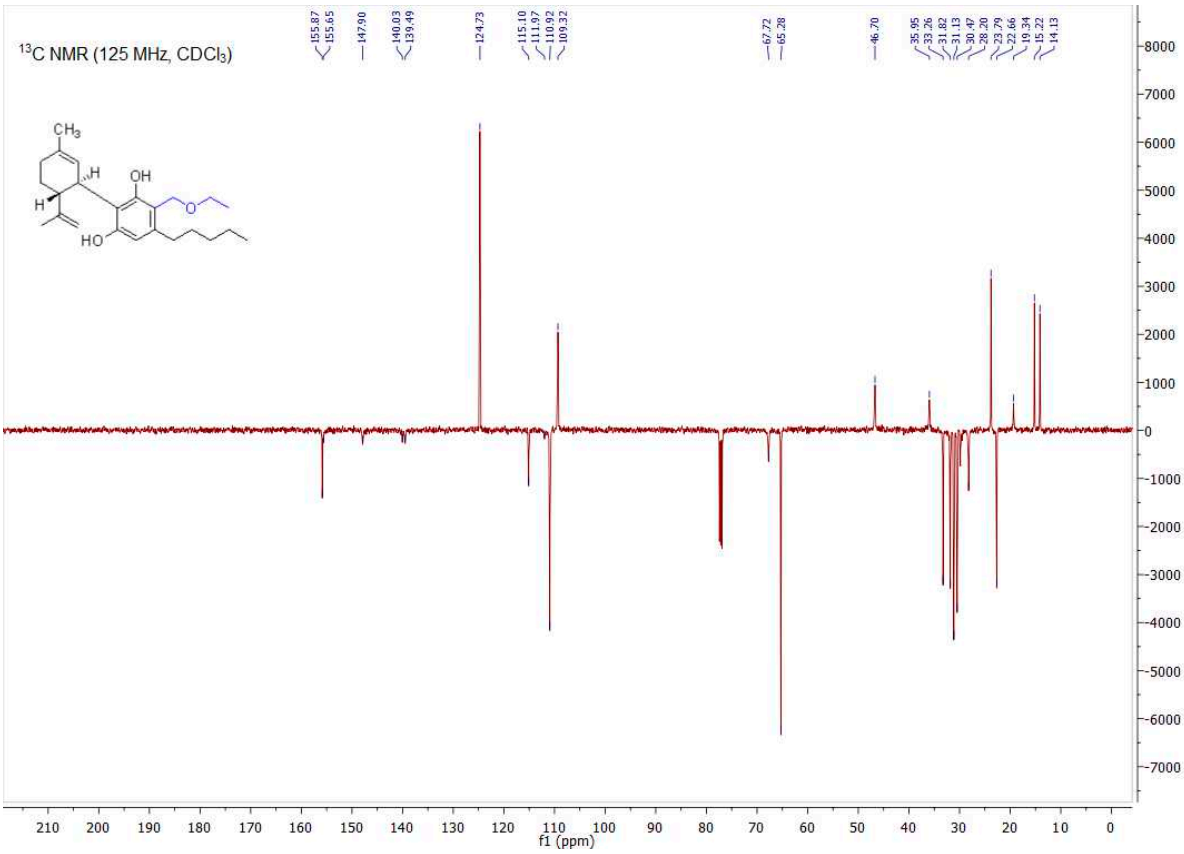
13C NMR (125 MHz, CDCl3)
155.87
155.65
147.90
140.03
139.49
124.73
115.10
111.97
110.92
109.32
67.72
65.28
46.70
35.95
33.26
31.82
31.13
30.47
28.20
23.79
22.66
19.34
15.22
14.13
CH3
H
OH
O
HO
f1 (ppm)

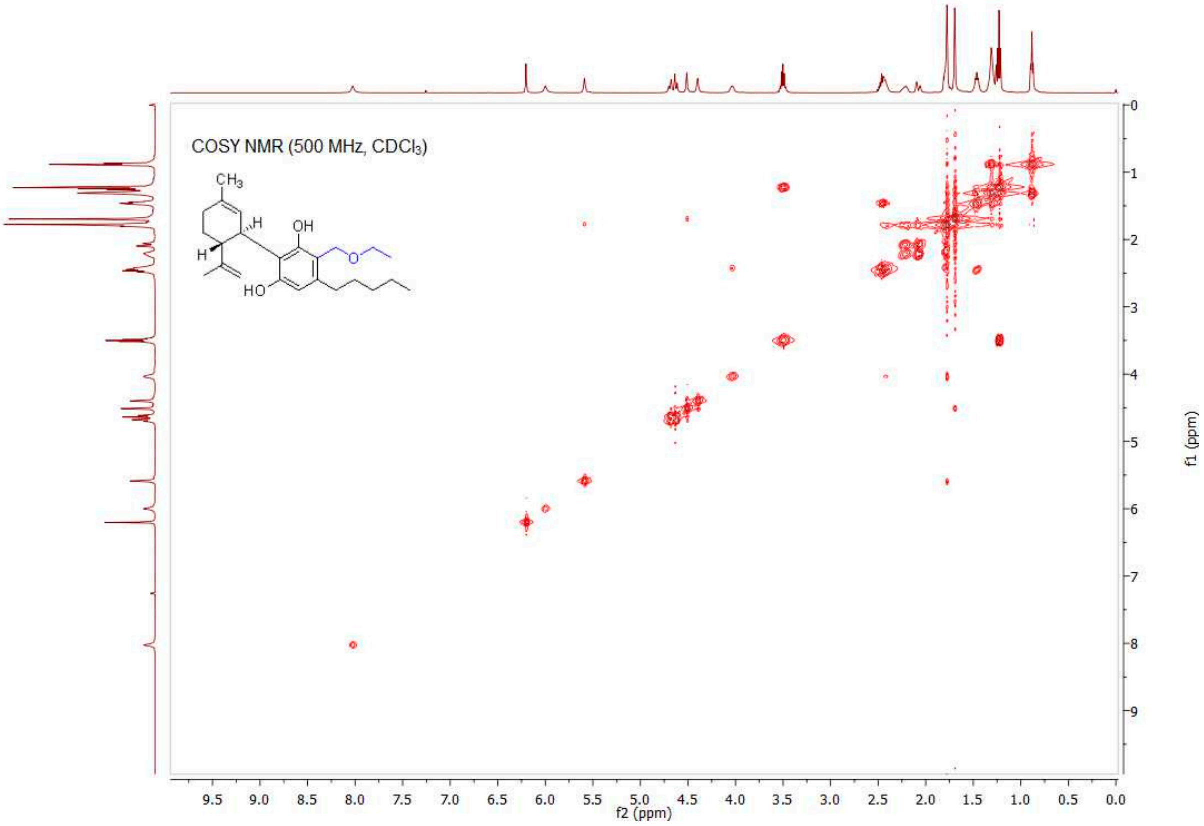
COSY NMR (500 MHz, CDCl3)
CH3
H
OH
O
HO
f2 (ppm)
f1 (ppm)

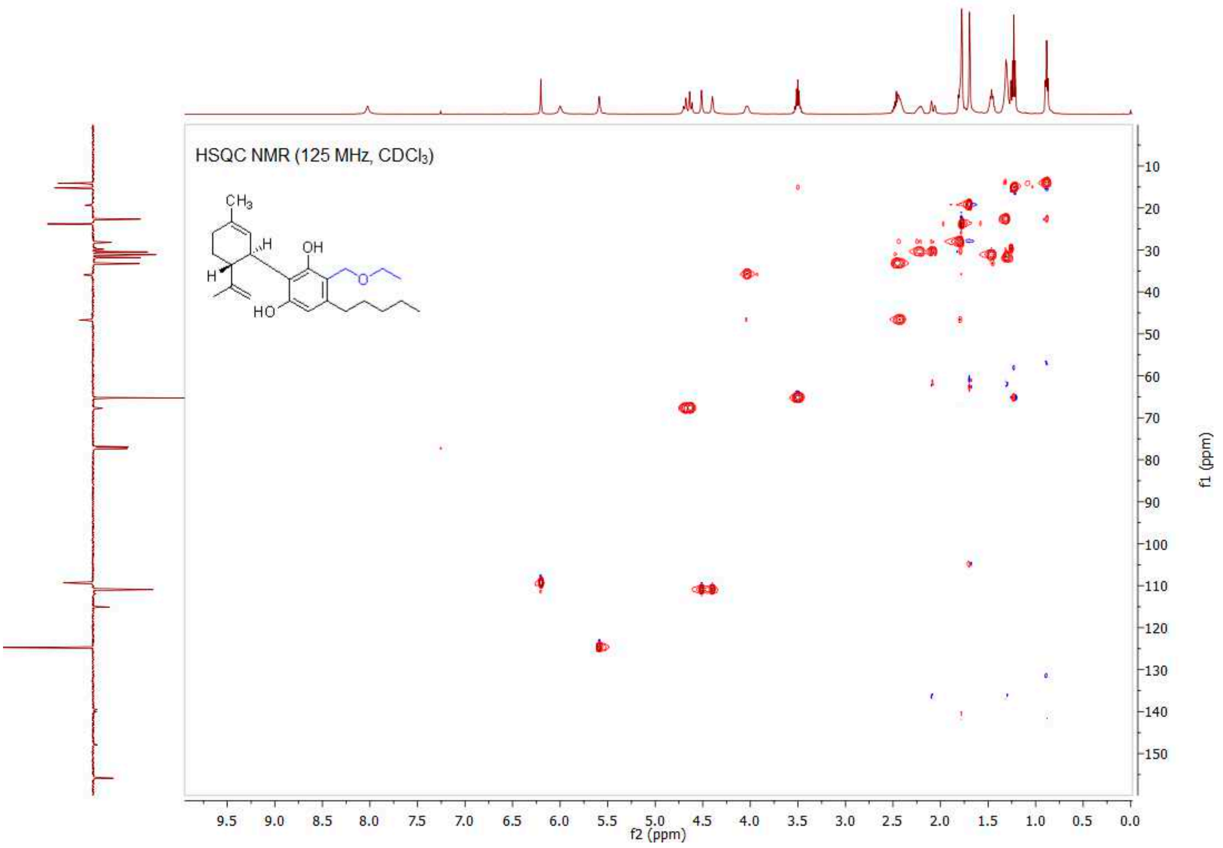

NMR spectra of CBD3

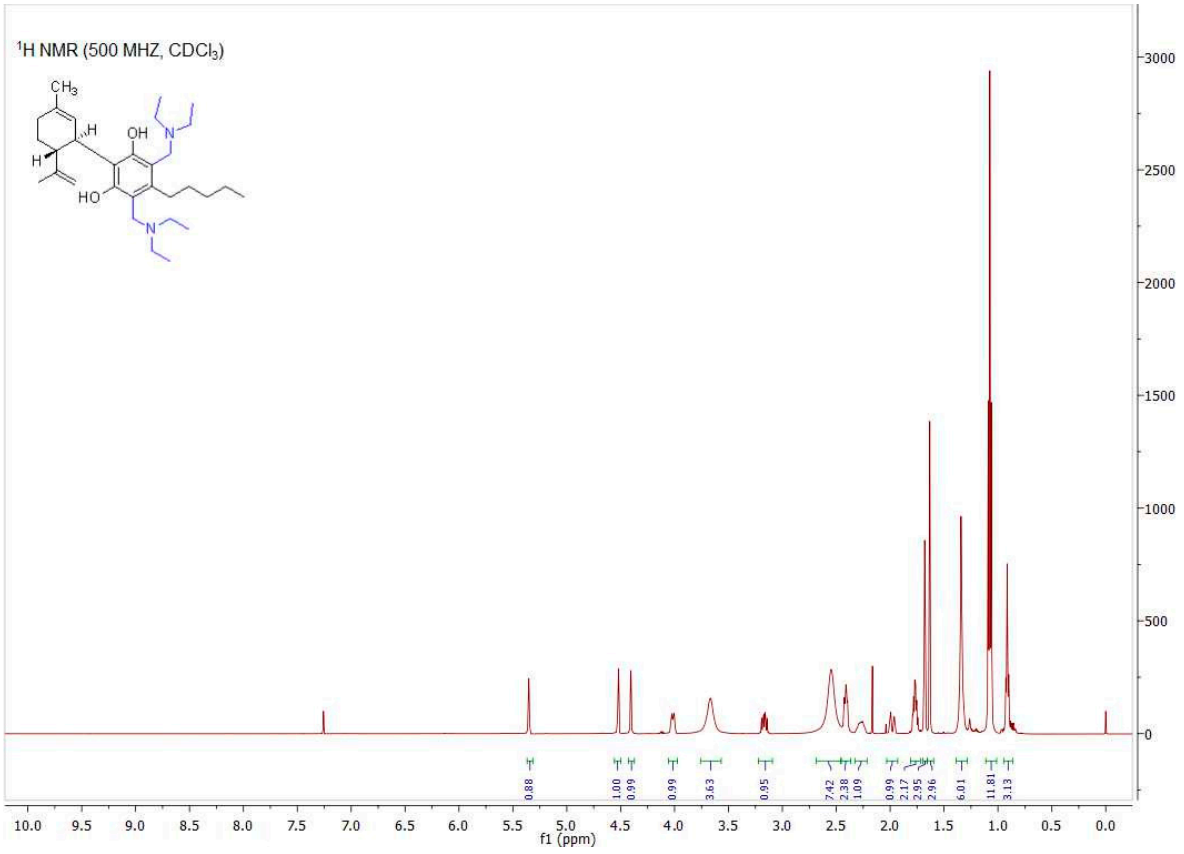

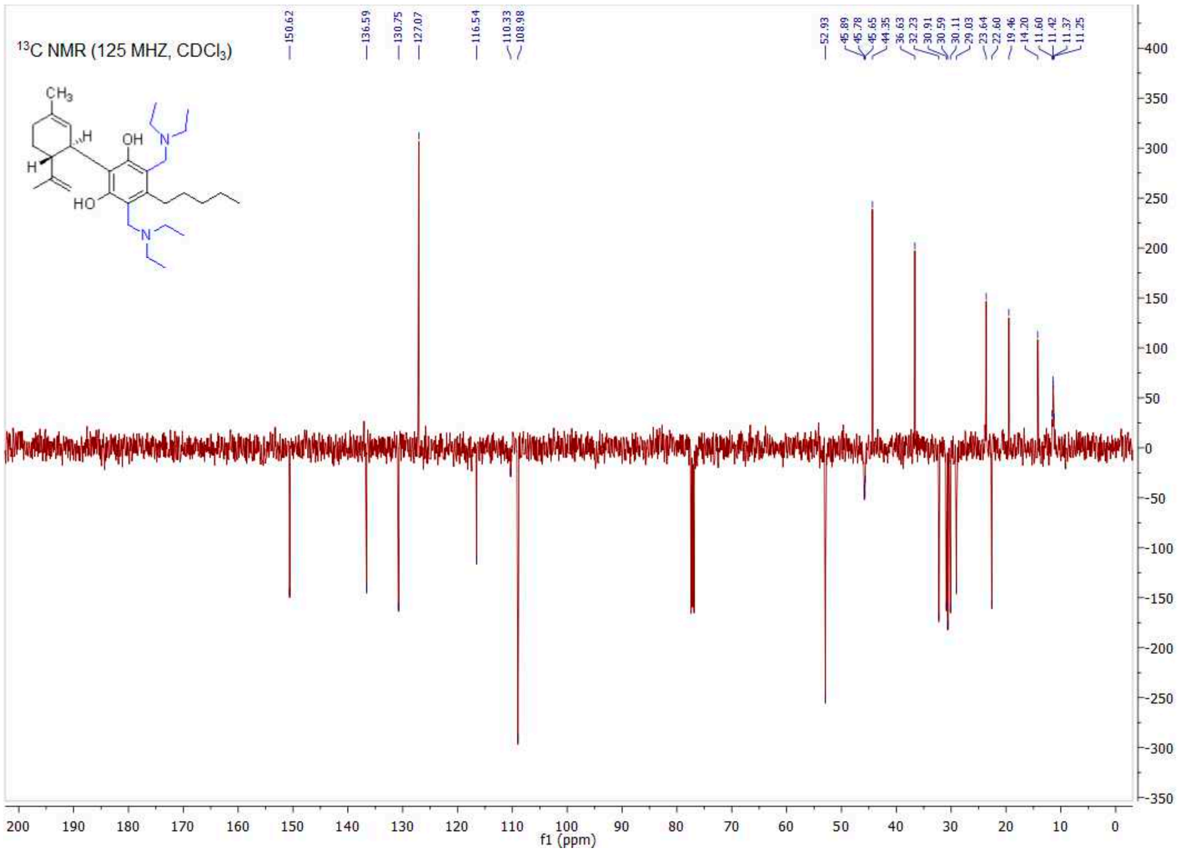

^{13}C NMR (125 MHZ, $CDCl_3$)
CH3
H
OH
N
H
HO
N
150.62
136.59
130.75
127.07
116.54
110.33
108.98
52.93
45.89
45.78
45.65
44.35
36.63
32.23
30.91
30.59
30.11
29.03
23.64
22.60
19.46
14.20
11.60
11.42
11.37
11.25
f1 (ppm)
